# Targeting central melanocortin receptors: a promising novel approach for treating alcohol abuse disorders

**DOI:** 10.3389/fnins.2014.00128

**Published:** 2014-06-03

**Authors:** Jeffrey J. Olney, Montserrat Navarro, Todd E. Thiele

**Affiliations:** ^1^Department of Psychology, University of North CarolinaChapel Hill, NC, USA; ^2^Bowles Center for Alcohol Studies, University of North CarolinaChapel Hill, NC, USA

**Keywords:** melanocortin, POMC, α-MSH, AgRP, MC3 receptor, MC4 receptor, ethanol

## Abstract

The melanocortin (MC) peptides are produced centrally by propiomelanocortin (POMC) neurons within the arcuate nucleus of the hypothalamus and act through five seven-transmembrane G-protein coupled melanocortin receptor (MCR) subtypes. The MC3R and MC4R subtypes, the most abundant central MCRs, are widely expressed in brain regions known to modulate neurobiological responses to ethanol, including regions of the hypothalamus and extended amygdala. Agouti-related protein (AgRP), also produced in the arcuate nucleus, is secreted in terminals expressing MCRs and functions as an endogenous MCR antagonist. This review highlights recent genetic and pharmacological findings that have implicated roles for the MC and AgRP systems in modulating ethanol consumption. Ethanol consumption is associated with significant alterations in the expression levels of various MC peptides/protein, which suggests that ethanol-induced perturbations of MC/AgRP signaling may modulate excessive ethanol intake. Consistently, MCR agonists decrease, and AgRP increases, ethanol consumption in mice. MCR agonists fail to blunt ethanol intake in mutant mice lacking the MC4R, suggesting that the protective effects of MCR agonists are modulated by the MC4R. Interestingly, recent evidence reveals that MCR agonists are more effective at blunting binge-like ethanol intake in mutant mice lacking the MC3R, suggesting that the MC3R has opposing effects on the MC4R. Finally, mutant mice lacking AgRP exhibit blunted voluntary and binge-like ethanol drinking, consistent with pharmacological studies. Collectively, these preclinical observations provide compelling evidence that compounds that target the MC system may provide therapeutic value for treating alcohol abuse disorders and that the utilization of currently available MC-targeting compounds- such as those being used to treat eating disorders- may be used as effective treatments to this end.

## Introduction and overview

The melanocortin (MC) system has been implicated in a host of physiological functions. Emerging evidence indicates that this neuropeptide system is also involved in modulating the neurobiological responses to drugs of abuse, including ethanol. This review will present notable discoveries in the preclinical realm that highlight the role of the central MC system in the neurobiological responses to ethanol. In light of these findings, special consideration will be given to the MC system as a potential target for treating alcohol use disorders (AUDs) in clinical populations.

**Figure 1:**
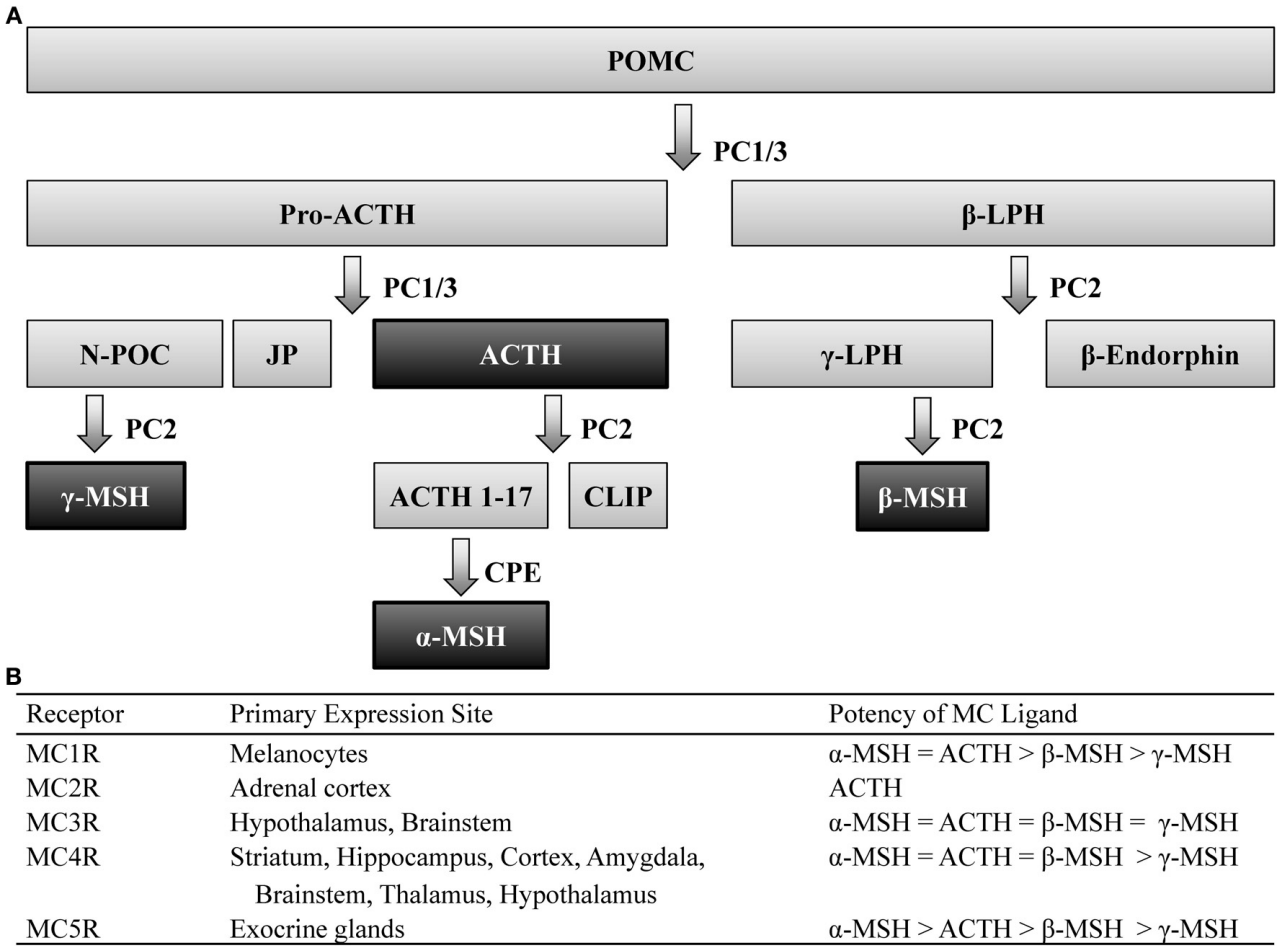
**Melanocortin ligands and receptors. (A)** Simplified model of posttranslational processing of POMC. Agonists that act at MCR1-5 are highlighted black. **(B)** These ligands exert their effects via five G-protein coupled receptors, differentially expressed throughout the body, with varying degrees of potency. ACTH, adrenocorticotropic hormone; CLIP, corticotrophin-like intermediate lobe peptide; CPE, carboxypeptidase E; JP, junctional peptide; LPH, lipotropic hormone; N-POC, *N*-pro-opiocortin; MC, melanocortin; MSH, melanocyte stimulating hormone; PC, prohormone convertase; POMC, proopiomelanocortin.

Post-translational processing of the prohormone, proopiomelanocortin (POMC), involves cleavage by two prohormone convertases, PC1/3 and PC2, that yield two different classes of peptides, melanocortins (MCs) and the opioid β-endorphin- hence the term pro-opio-melanocortin. Of the MC family, POMC cleavage leads to several peptides including: α-, β-, and γ-melanocyte stimulating hormone (MSH) as well as adrenocorticotrophic hormone (ACTH), which all share the same core amino acid sequence, His-Phe-Arg-Trp, that is required for the biological activity of these peptides. POMC is primarily expressed within the central nervous system (CNS) within the nucleus of the solitary tract (NST) of the brainstem, the arcuate nucleus of the hypothalamus (Arc), and the pituitary (Joseph et al., 1983; Hadley and Haskell-Luevano, 1999). Peripheral expression of POMC has also been observed in the skin, spleen, thyroid, and the gastrointestinal tract (Smith and Funder, 1988). The post-translational processing of POMC is tissue specific (Pritchard et al., 2002), which allows a multitude of peptides to be derived from a single prohormone (Figure 1).

These peptides exert their actions onto five MC receptors (MCRs). These five receptors, numbered MC1-5R, which represent the order in which they were cloned, are seven-transmembrane G-protein coupled receptors coupled through Gα_s_ signaling pathways. The MC1R, MC2R, and MC5R have been observed to be widely expressed throughout peripheral tissue (see Chhajlani, 1996; Wikberg, 1999 for review). Although the MC3R and MC4R have also been observed in the periphery (Gantz et al., 1993a; Chhajlani, 1996), these MCRs are the predominant MCR subtypes expressed in the brain (Mountjoy, 2010) and thus the MC3R and MC4R will be the focus of this review.

Immunohistochemical (IHC) and *in situ* hybridization (ISH) studies have localized central expression of the MC4R to be widely distributed across the striatum, hippocampus, cortex, amygdala, brainstem, thalamus, and hypothalamus (Mountjoy et al., 1994; Kishi et al., 2003; Liu et al., 2003). Similar studies have determined that expression of the MC3R overlaps a great deal with the MC4R, although the former is restricted to the hypothalamus and, to a lesser extent, the brainstem (Roselli-Rehfuss et al., 1993). It is also worth noting that despite their overlapping expression patterns, these MCR subtypes possess differential affinity for MC peptides (Figure 1). Notably, the four MC peptides are approximately equipotent at the MC3R (Roselli-Rehfuss et al., 1993) though γ-MSH is reported to possess much greater selectivity and potency at the MC3R than at any other MCR subtype (Renquist et al., 2011). Conversely, α-MSH, β-MSH, and ACTH are similarly potent for the MC4R while γ-MSH is the least (Gantz et al., 1993b). Importantly, γ-MSH has a much greater affinity and potency for the MC3R than MC4R (Grieco et al., 2000; Kask et al., 2000)- a fact that has been taken advantage of when attempting to selectively target MCR subtypes (Marks et al., 2006; Lee et al., 2008).

In addition to the aforementioned MC peptides and receptors, the MC system is unique among neuropeptide systems in that an endogenous antagonist, agouti-related protein (AgRP) exists for the system. AgRP has been found to be predominately expressed in the Arc in the CNS as well as the adrenal cortex in the periphery (Broberger et al., 1998; Haskell-Luevano et al., 1999). Although AgRP is synthesized within the Arc, it has widespread projections to other hypothalamic subnuclei including the paraventricular nucleus of the hypothalamus (PVN), ventromedial hypothalamus (VMH), dorsomedial hypothalamus (DMH), and the lateral hypothalamus (LH) as well as regions beyond the hypothalamus including the NST, amygdala, bed nucleus of the stria terminalis (BNST), ventral tegmental area (VTA), and nucleus accumbens (NAc; Broberger et al., 1998; Bagnol et al., 1999). This peptide acts as a competitive antagonist that is equipotent at both MC3R and MC4R. Interestingly, evidence also exists that suggests AgRP acts as an inverse agonist- reducing adenylyl cyclase activity to below baseline levels (Nijenhuis et al., 2001). This presents the intriguing possibility that AgRP may be able to regulate MCR activity independent of endogenous MC peptide activity.

Given the multitude of peptides derived from the POMC prohormone, it comes as no surprise that the MC system has been implicated in a myriad of physiological functions. The term melanocortin was coined when early research found that the peptide exhibited melanotropic and/or adrenocorticotropic activity. The MC system has since been implicated in a wide array of other functions, such as sexual behavior, inflammation, and feeding behavior. Although these functions have been discussed in more detail elsewhere (see Gantz and Fong, 2003 for review), this review focuses on the emerging evidence that the MC system significantly modulates the neurobiological responses to ethanol.

## Ethanol's effects on the melanocortin system

Through an ever-increasing body of literature, it is clear that ethanol causes significant alterations in the MC system. The first hint that the MC system was involved in the neurobiological responses to ethanol was observed when ethanol exposure caused a marked reduction in hypothalamic levels of ACTH, one of the precursors to α-MSH (Gambert et al., 1981). Wilkinson et al. (1986) later reported similar reductions in α-MSH levels following exposure to acute ethanol vapor in mice.

These reports and many others demonstrate that ethanol impacts the MC system; however, it appears that the manner in which ethanol is exposed to the subject can determine how the MC system is altered (see Table 1). For example, increases in POMC levels have been observed following ethanol exposure *in vitro* (Pastorcic et al., 1994) while Kokare et al. (2008) reported that rats in a withdrawal state following chronic exposure to an ethanol-liquid diet displayed similar increases in α-MSH. However, chronic ethanol exposure via vapor chamber, and in the absence of withdrawal, has been shown to decrease POMC levels in rats (Scanlon et al., 1992). Similarly, chronic exposure to an ethanol liquid-diet without withdrawal also reduced levels of POMC as well as convertase PC1/3 (Navarro et al., 2013). Given that PC1/3 is an enzyme responsible for cleaving POMC into subsequent MC peptides, it should come as no surprise that chronic ethanol exposure via an ethanol liquid-diet has been shown to cause a reduction in α-MSH as well (Rainero et al., 1990; Navarro et al., 2008).

**Table 1:** **Summary of findings examining the effects of ethanol exposure on the melanocortin system**.

**Method of ethanol delivery**	**Brain area**	**Alterations in protein expression**
Acute ethanol diet	Amygdala	= α-MSH^6^
	Arc	↓ α-MSH^6^, = AgRP^4^, ↓ POMC^5^, ↓ PC1/3^5^, = PC2^5^
	BNST	↓ α-MSH^4^
	CeA	= α-MSH^4^, ↓ α-MSH^3^
	DMH	= α-MSH^4^, ↓ α-MSH^3^
	Frontal cortex	= α-MSH^6^
	Hippocampus	= α-MSH^6^
	LH	= α-MSH^4,6^
	NAc	= α-MSH^6^
	PAG	= α-MSH^4^
	Pituitary	↓ α-MSH^6^
	PVN	= α-MSH^4^
	PVT	↓ α-MSH^4^
	Striatum	= α-MSH^6^
	Substantia nigra	↓ α-MSH^6^
Chronic ethanol diet	Arc	↓ α-MSH^3^, ↓POMC^5^
	BNST	↓ α-MSH^4^
	CeA	↑ α-MSH^3^, ↓ α-MSH^4^
	DMH	↑ α-MSH^3^, = α-MSH^4^
	LH	= α-MSH^3^, ↓ α-MSH^4^
	PAG	= α-MSH^4^
	PVN	↑ α-MSH^3^, = α-MSH^4^
	PVT	= α-MSH^3^, ↓ α-MSH^4^
Withdrawal from ethanol diet	Arc	↑ α-MSH^3^
	CeA	↑ α-MSH^3^
	DMH	↑ α-MSH^3^
	LH	= α-MSH^3^
	PVN	↑ α-MSH^3^
	PVT	= α-MSH^3^
Vapor chamber	Hypothalamus	↓ POMC^7^
	Pituitary	↓ α-MSH^8^
Voluntary consumption	Hypothalamus	↑ POMC^2^
Bath application (*in vitro*)	Hypothalamus	↑ POMC^1^

↑, peptide/protein levels increase in response to ethanol exposure; ↓, peptide/protein levels decrease in response to ethanol exposure; =, ethanol has no effect on peptide/protein levels. AgRP, agouti-related protein; Arc, arcuate nucleus of the hypothalamus; BNST, bed nucleus of the stria terminalis; CeA, central nucleus of the amygdala; DMH, dorsomedial hypothalamus; LH, lateral hypothalamus; MSH, melanocyte stimulating hormone; NAc, nucleus accumbens; PAG, periaqueductal gray; POMC, proopiomelanocortin; PVN, paraventricular nucleus of the hypothalamus; PVT, paraventricular nucleus of the thalamus. References are as follows:

1 Cubero et al. (2010);

2 De Waele and Gianoulakis (1994);

3 Kokare et al. (2008);

4 Navarro et al. (2008);

5 Navarro et al. (2013);

6 Rainero et al. (1990);

7 Scanlon et al. (1992);

8 Wilkinson et al. (1986).

Additionally, a concerted effort has been made to explore the innate differences in the MC system of rodents that display enhanced ethanol consumption, which may be used to determine possible biomarkers to help identify individuals at risk of developing AUDs. To this end, De Waele and Gianoulakis (1994) observed a marked increase in POMC levels following voluntary consumption of ethanol in ethanol-preferring C57BL/6J (C57), but not ethanol-avoiding DBA/2 mice. Although these data are contradictory to many of the studies discussed above, the different methodologies (e.g., strain, species, modality of ethanol exposure, etc.) may, in part, account for these incongruities. Additionally, Lindblom et al. (2002) reported that ethanol-naïve Alko Alcohol (AA) rats, which are selectively bred to prefer ethanol, displayed elevated POMC expression and reduced AgRP mRNA in the Arc relative to their non-ethanol preferring ANA counterparts. The authors then went on to explore facets of the MC system beyond the peptides themselves and discovered that AA rats exhibited abnormal expression patterns of the MC3R in the shell of the NAc, PVN, VMH, and Arc. Moreover, a recent study from our lab found that an acute injection of ethanol causes a significant increase in AgRP levels within the Arc of C57 mice while the same effect was absent in low drinking 129/SvJ mice (Cubero et al., 2010). Together, these findings suggest that the different drinking behaviors between these animals may be attributed to innate differences in the functionality and responses to ethanol by the MC system, although further investigation is required in order to precisely determine the neurobiological locus responsible for the differences between these strains.

## The effects of the melanocortin system on ethanol consumption

Several lines of evidence have indicated that the MC system plays a rather important role in modulating the neurobiological responses to ethanol (see Table 2). Initial evidence that the MC system modulates ethanol responding can be credited to Ploj et al. (2002) when they demonstrated that intracerebroventricular (i.c.v.) infusions of the potent, non-selective MCR agonist, melanotan-II (MTII), significantly reduced voluntary ethanol consumption in AA rats. Similarly, both central and peripheral injections of MTII resulted in significant reductions in ethanol consumption in C57 mice (Navarro et al., 2003). Together, these studies indicate that the MC system serves to protect against ethanol consumption.

**Table 2:** **Summary of findings targeting the melanocortin system on ethanol-related phenotypes**.

**MCR examined**	**Notable findings**
Non-selective MCR	MTII (i.c.v.) ↓ voluntary consumption in AA rats^11^
	MTII (i.c.v.) ↓ voluntary consumption in C57 mice^6^
	Intra-amygdala MTII ↓ voluntary consumption in P rats^12^
	α-MSH (i.c.v.) ↓ ethanol-induced anxiolysis^2^
	γ-MSH (i.c.v.) ↓ ethanol-induced anxiolysis^1^
	γ-MSH (i.c.v.) ↑ withdrawal-induced anxiogenesis^1^
	α-MSH (i.c.v.) ↓ ethanol-induced antidepressive-like effects^3^
MC3R	MTII (i.c.v.) ↓ voluntary consumption in MC3R^−/−^ mice^7^
	MTII (i.c.v.) ↓ binge-like ethanol drinking in both MC3R^+/+^ and MC3R^−/−^ mice, but was more effective in MC3R^−/−^ mice^10^
MC4R	Selective MC4R agonist (i.c.v.) ↓ voluntary consumption in C57 mice^7^
	MTII (i.c.v) did not alter voluntary consumption in MC4R^−/−^ mice^9^
	Selective MC4R agonist directly infused into the NAc ↓ voluntary consumption in rats^4^
	Selective MC4R agonist directly infused into the NAc ↓ ethanol palatability in rats^5^
	Selective MC4R agonist (i.c.v.) ↑ ethanol-induced anxiolysis^2^
	Selective MC4R agonist (i.c.v.) ↓ withdrawal-induced anxiogenesis^2^
	Selective MC4R agonist (i.c.v.) ↑ ethanol-induced antidepressive-like effects^3^
	Selective MC4R agonist (i.c.v.) ↓ withdrawal-induced depressive-like symptoms^3^
AgRP	AgRP (i.c.v.) blocked MTII-induced ↓ voluntary consumption in C57 mice^6^
	AgRP (i.c.v.) ↑ voluntary consumption in C57 mice^7^
	AgRP^−/−^ display ↓ voluntary ethanol consumption, ↓ operant ethanol seeking, ↓ binge-like ethanol drinking^8^

↑, increase; ↓, decrease. AA, Alko Alcohol; AgRP, agouti-related protein; C57, C57BL/6J; LH, lateral hypothalamus; MSH, melanocyte stimulating hormone; MTII, melanotan-II; NAc, nucleus accumbens; P, alcohol-preferring; i.c., intracisternal; i.c.v., intracerebroventricular. References are as follows:

1 Jansone et al. (2009);

2 Kokare et al. (2006);

3 Kokare et al. (2008);

4 Lerma-Cabrera et al. (2012);

5 Lerma-Cabrera et al. (2013);

6 Navarro et al. (2003);

7 Navarro et al. (2005);

8 Navarro et al. (2009);

9 Navarro et al. (2011);

10 Olney et al. (2014);

11 Ploj et al. (2002);

12 York et al. (2011).

Although these findings indicate that the MCR signaling is inversely related to ethanol consumption, the non-selective nature of these MC agents prevented the ability to pinpoint which receptor subtype are mediating these effects. With the advent of mutant mice lacking these specific receptors, researchers have begun to elucidate the specific contribution of the individual MCR subtypes to ethanol consumption focusing on the two subtypes predominately expressed in the CNS: the MC3R and MC4R. Using mutant mice deficient in MC3R (MC3R^−/−^) and the non-selective MCR agonist, MTII, Navarro et al. (2005) sought to determine the contribution of the specific MCR subtypes. With this approach, if the MC3R plays a significant role in modulating the protective effects of MTII, then treatment with the agonist should reduce ethanol consumption in wild-type (MC3R^+/+^), but not MC3R^−/−^ mice. However, it was found that MTII significantly reduced 24-h voluntary ethanol consumption in both MC3R^+/+^ and MC3R^−/−^ mice. This finding suggested that the MC3R did not contribute to the protective effects of MTII, leaving MC4R as the logical mediator of this effect. To test this, the authors then treated standard C57s with the selective MC4R agonist, *cyclo*(NH-CH2-CH2-CO-His-_D_-Phe-Arg-Trp-Glu)-NH2, and found that it caused a dose-dependent reduction in ethanol intake. Consistent with these findings, this same group later conducted a similar study with MC4R^−/−^ mice and found that treatment with MTII significantly blunted ethanol intake in MC4R^+/+^, but not MC4R^−/−^ mice (Navarro et al., 2011). Interestingly, the authors also noted that peripheral injections of MTII significantly reduced ethanol consumption in both genotypes, which suggests that other MCRs in the periphery may participate in the protective effects of MTII. Taken together, these studies suggest that central MC4R, but not MC3R, contribute to blunted ethanol consumption.

Despite these findings that suggest the MC3R does not play a role in ethanol consumption, a number of previous studies have indicated that this receptor may serve as an inhibitory autoreceptor on POMC neurons (see Renquist et al., 2011 for review). Briefly, the MC3R has been found to be expressed on POMC neurons (Bagnol et al., 1999) and activation of these receptors using the selective MC3R agonist, _D_-Trp^8^-γ-MSH, increased IPSC frequency on these neurons (Cowley et al., 2001) and causes a marked reduction in POMC mRNA levels in rats (Lee et al., 2008). Consistent with these findings, selective activation of MC3Rs also results in enhanced food consumption (Lee et al., 2008)- an effect that is notably absent in MC3R^−/−^ mice (Marks et al., 2006).

Given the converging evidence from electrophysiological, anatomical, and behavioral studies that indicate MC3R acts as an inhibitory autoreceptor on POMC neurons, our lab decided to revisit the MC3R and further probe its role in binge-like ethanol consumption in MC3R^−/−^ mice following a range of doses of MTII. Consistent with previous results (Navarro et al., 2005), we found that central infusions of MTII significantly reduced binge-like ethanol consumption in both MC3R^−/−^ and MC3R^+/+^ mice during the first hour of testing (Olney et al., 2014). Interestingly, however, we also observed that MC3R^−/−^ mice were more sensitive to the protective effects of MTII as all doses of MTII tested produced a significant reduction in binge-like ethanol consumption while only the highest dose significantly attenuated consumption in MC3R^+/+^ mice. Thus, although the MC4R may be the main effector of the protective effects of MTII against ethanol consumption, this data suggests that the MC3R may contribute to ethanol consumption as a pre-synaptic inhibitory autoreceptor on POMC neurons by regulating endogenous α-MSH signaling onto MC4Rs, or possibility by directly opposing the actions of the MC4R post-synaptically.

Together, these studies demonstrate that MC agents are capable of modulating the neurobiological responses to ethanol. However, it is worth noting that these effects are not specific to ethanol intake. Indeed, a single i.c.v. infusion of MTII causes significant reductions in ethanol, food, sucrose, and saccharin, but not water intake (Navarro et al., 2011)- suggesting that MC agonists reduce consumption of salient reinforcers, regardless of caloric content. Moreover, recent findings have also implicated the MC system in other neurobiological responses to ethanol beyond intake. It is well established that ethanol use is associated with anxiety and depression (Roelofs, 1985; Grant and Harford, 1995; Gilman and Abraham, 2001). Interestingly, MC agonists appear to suppress the anxiolytic and antidepressive-like properties of acute ethanol while antagonists enhanced these effects (Kokare et al., 2006, 2008; Jansone et al., 2009). What is more, these same studies also reported MC agonists enhance the anxiogenic effects and depressive-like symptoms of ethanol withdrawal while antagonists had the reverse effect. It has been postulated that these findings may be due to the neurobiological effects of ethanol on MC signaling within the CeA- an area that has been identified to be critically involved in depression and anxiety (Kask and Schiöth, 2000; Huang and Lin, 2006). In fact, ethanol has been observed to alter α-MSH expression within the CeA (Kokare et al., 2008) that coincides with the anxiolytic and anxiogenic properties of acute ethanol and withdrawal, respectively (see Table 1). Altogether, these findings indicate that the MC system may modulate numerous neurobiological responses to ethanol use beyond consumption.

The studies outlined above were successful in elucidating the role of specific subtypes of MCRs in ethanol consumption, but they were unable to determine which brain regions convey these effects. One recent study sought to determine the participation of MCRs within the amygdala in regulating ethanol consumption in alcohol-preferring (P) rats (York et al., 2011). Following site-directed infusion of MTII into the amygdala, P rats exhibited a significant reduction in 24-h ethanol consumption. Interestingly, the authors also noted treatment with the non-selective MC3/4R antagonist, SHU9119, produced a similar reduction in ethanol intake among P rats despite the fact that MCR antagonists have previously been found to augment ethanol drinking (see below). However, the authors also reported that treatment with SHU9119 resulted in an increase in 24-h water intake in these animals; therefore, the observed reduction in ethanol intake following inhibition of central MCRs may be due to the fact that the animals replaced ethanol intake with water drinking during a state of increased feeding behavior. Additionally, direct infusion of a selective MC4R agonist into the VTA and NAc, but not the LH, significantly reduced ethanol consumption in rats (Lerma-Cabrera et al., 2012). Later studies by the same group revealed that similar activation of MC4Rs within the NAc, but not the LH, decreased the palatability of ethanol in rats (Lerma-Cabrera et al., 2013). Specifically, the authors noted that treatment with a selective MC4R agonist directly into the NAc significantly decreased the duration of hedonic responses and increased the frequency of aversive responses following delivery of ethanol via an intraoral cannula. This latter study raises the intriguing possibility that one mechanism by which the MC system regulates ethanol consumption is by modulating the subjective orosensory responses to ethanol, which has previously been reported to influence ethanol consumption (Brasser et al., 2012).

Given that a wealth of research has demonstrated that MCR signaling regulates ethanol consumption and that POMC and AgRP circuits exert oppositional effects on feeding behavior, it should come as no surprise that modulation of AgRP has been reported to alter ethanol consumption as well. Indeed, central infusion of the active AgRP fragment, AgRP-(83-132), was found to significantly augment ethanol drinking in mice (Navarro et al., 2005). Furthermore, these authors also demonstrated that pretreatment with AgRP-(83-132) blocked the ability of MTII to attenuate ethanol consumption- providing further confirmation that this peptide regulates ethanol consumption via antagonistic actions at MCRs. Moreover, deletion of the gene that encodes for AgRP produces a mouse that exhibits blunted responding to ethanol in a variety of paradigms relative to wild-type controls (Navarro et al., 2009). Specifically, these AgRP^−/−^ mice displayed reduced ethanol-reinforced lever-pressing behavior as well as reduced consumption in a two-bottle choice and binge-like drinking paradigm. Together these data indicate that AgRP regulates ethanol consumption by functionally opposing MCR signaling.

Although the research involving AgRP thus far has been limited to its capacity as an MCR antagonist, recent evidence proposes the exciting possibility that AgRP may act beyond MCRs. Dietrich et al. (2012) recently reported that AgRP neurons were found to modulate reward processing as well as contribute to plastic changes in reward circuitry. Specifically, the authors observed that impairment of AgRP neurons increased cocaine-induced conditioned place preference (CPP) and novelty seeking- a behavior known to be associated with dopamine (DA) levels (Bradberry et al., 1991; Hooks et al., 1991; Zald et al., 2008). The authors then went on to demonstrate that mice with impaired AgRP neurons exhibited increased levels of DA and enhanced long-term potentiation. This study represents the first demonstration that AgRP neurons may function in a dimension beyond regulating POMC signaling. Although pharmacological MCR agents were observed to be ineffective at modulating the effects observed in this report, it is important that one does not hastily conclude that this provides definitive evidence that the AgRP peptide acts beyond MCRs. Importantly, the authors impaired AgRP circuitry via neuronal ablation or genetic knockdown of neuronal activity. Indeed, AgRP neurons are known to coexpress neuropeptide Y (NPY) as well as GABA (Shutter et al., 1997; Hahn et al., 1998) and, given that depressed AgRP circuitry appeared to decrease the inhibitory tone on DAergic VTA neurons, the effects observed here are most likely due to GABA- although future studies may seek to clarify the mechanism governing these observed effects.

## Targeting the melanocortin receptors to treat alcohol abuse disorders

Progress in our understanding of the MC system's involvement in the neurobiological effects of ethanol has made great strides using preclinical models. As such, the next logical step is to apply our understanding to develop effective treatments for clinical populations suffering from AUDs. Perhaps the most widely studied function of the central MC system is its role in feeding behavior (see Ellacott and Cone, 2006 for review). This is of importance when one considers the fact that several lines of evidence suggest that both food and alcohol consumption are governed by shared pathways (Thiele et al., 2003; Volkow et al., 2013). Indeed, ethanol is unique among the drugs of abuse in that, like food, it holds caloric value- thereby providing a potential source of nutrients- and has an exclusive oral route of administration. Moreover, a multitude of clinical reports have described a relatively high rate of co-morbidity between alcohol abuse and eating disorders (Higuchi et al., 1993; Holderness et al., 1994; Dansky et al., 2000; Sinha and O'Malley, 2000; Anderson et al., 2005), which should come as no surprise considering aberrant consummatory behavior and a loss of control are hallmarks of both conditions. What is more, it has previously been documented that comorbid patients often show improvements in both conditions when receiving treatment for one disorder (Dawe and Staiger, 1998; Daniels et al., 1999; O'Malley et al., 2007). Fortunately, clinical research studies are already underway that are investigating drugs that target the central MC system for the treatment of eating disorders and energy balance.

One compound currently undergoing such investigations is MSH/ACTH(4-10), an MC4R agonist that, when administered intranasally (i.n.), has been shown to gain access to the CNS (Born et al., 2002) where it may serve to augment central MC functioning. Studies with this compound have demonstrated that participants treated with an i.n. dose of MSH/ACTH(4-10) display significantly increased lipolysis (Wellhöner et al., 2012) and reduced adiposity (Fehm et al., 2001). Another compound, the orally active MC4R agonist, MK-0493, has similarly been investigated as a pharmacological treatment for weight loss in obese patients. Krishna et al. (2009) recently reported that patients treated daily with MK-4093 exhibited a small, albeit non-significant, reduction in food consumption over the course of 24-h. Although this drug was found to be only marginally successful, it is worth noting that some pharmacological treatments have been found to be more effective when the treatment program is supplemented with some forms of behavioral therapy (Anton et al., 2006). Therefore, MK-4093 alone may have been insufficient at treating excessive food consumption, but its effectiveness as part of a more complete, multidimensional treatment program should not be dismissed.

In addition to supplementing behavioral therapy, these MC compounds may be used to in tandem with other pharmacological treatments to achieve a more effective treatment strategy. These combinatorial pharmacological therapies offer unique benefits over traditional monotherapies in that the two compounds may produce additive effects- or may even work synergistically with one another. Indeed, opioid signaling has been shown to inhibit the activity of POMC neurons (Kelly et al., 1990; Cowley et al., 2001). As such, perhaps the most impressive data using this strategy comes from studies using naltrexone, an already FDA approved drug used to treat opioid and ethanol dependence (Bouza et al., 2004), in tandem with bupropion, another FDA approved medication for depression (Davidson and Connor, 1998), smoking cessation (Roddy, 2004), and weight-loss (Anderson et al., 2002) that has been shown to stimulate MC pathways (Greenway et al., 2009b). The combination of naltrexone and bupropion has already garnered a great deal of interest as an effective treatment regimen for obesity. Indeed, a number of reports are available that demonstrate improved efficacy of a combination of naltrexone and bupropion over a treatment regimen that uses either drug alone (Greenway et al., 2009a,b; Apovian et al., 2013; Hollander et al., 2013; McElroy et al., 2013; Smith et al., 2013). Furthermore, a combination of naltrexone and bupropion used in conjunction with behavioral therapy was found to be a highly effective treatment regimen for weight loss (Wadden et al., 2011).

Like most pharmacological treatment options, compounds that target the MC system are not without their side-effects. The most common adverse side-effects reported in patients participating in clinical trials are nausea and headache (Greenway et al., 2009a,b; Apovian et al., 2013; McElroy et al., 2013; Smith et al., 2013). Despite these adverse side-effects, these studies report these compounds to be largely well-tolerated by the patients- though further examination of the safety and tolerability of these compounds is encouraged as these drugs continue through clinical trials. Additionally, though the MC system is involved in a wide range of physiological functions, some of the non-specific effects of these compounds may be beneficial toward the ultimate goal of treating AUDs. For example, the utility of the fact that MC compounds act on neurobiological circuits involved in feeding behavior has already been discussed- suggesting this form of treatment may be most beneficial for these patients with comorbid eating and alcohol abuse disorders. What is more, it is well established that the activation of MCR pathways exert potent inhibitory control of central inflammatory processes (see Muceniece and Dambrova, 2010 for review). Numerous reports have demonstrated that anti-inflammatory agents produce a marked reduction in ethanol consumption (Agrawal et al., 2011; McIver et al., 2012) while pro-inflammatory agents increase consumption (Blednov et al., 2011). Therefore, the anti-inflammatory effects of the MC system, like its effects on feeding behavior, may provide another avenue by which these MC compounds may act to effectively treat AUDs.

Together, these findings clearly illustrate the utility of MC compounds in treating such neuropsychological disorders- whether it be alone or in concert with other behavioral or pharmacological treatments. Interestingly, the development of pharmacological interventions aimed at treating eating disorders has received considerable attention from drug developers over recent years (Mancini and Halpern, 2006). Given the common and overlapping neurobiological pathways shared between alcohol abuse and eating disorders and the effectiveness across conditions that these treatments have demonstrated, further investigations of the efficacy of these drugs in the development for eating disorders- such as those mentioned above- in treating AUDs may prove to be a worthwhile endeavor.

## Conclusion

For more than a decade, significant progress has been made in elucidating the role of the MC system in the neurobiological responses to ethanol. Indeed, ethanol has been shown to significantly alter the functionality of the central MC system (see Table 1) and that compounds that target the MC system can protect against ethanol consumption and other ethanol-related behaviors (see Table 2). Together, these findings hold promise for the MC system as a potential target for therapeutic intervention for AUDs. In fact, a number of therapeutic drugs targeting the MC system are currently under development for other clinical disorders. Given the commonalities between AUDs and other disorders- such as eating disorders- the utilization of these available drugs as potential means to alleviate the symptoms associated with alcohol use and abuse may be a beneficial avenue to pursue in the future.

### Conflict of interest statement

The authors declare that the research was conducted in the absence of any commercial or financial relationships that could be construed as a potential conflict of interest.
